# From computational screening to the synthesis of a promising OER catalyst†

**DOI:** 10.1039/d4sc00192c

**Published:** 2024-06-11

**Authors:** Sai Govind Hari Kumar, Carlota Bozal-Ginesta, Ning Wang, Jehad Abed, Chung Hsuan Shan, Zhenpeng Yao, Alan Aspuru-Guzik

**Affiliations:** a Department of Chemistry, University of Toronto Toronto Canada saigovind.harikumar@mail.utoronto.ca; b Department of Computer Science, University of Toronto Toronto Canada; c Catalonia Institute for Energy Research Barcelona Spain; d Department of Materials Science and Engineering, University of Toronto Toronto Canada; e Department of Electrical and Computer Engineering, University of Toronto Toronto Canada; f Center of Hydrogen Science, Shanghai Jiao Tong University Shanghai China; g State Key Laboratory of Metal Matrix Composites, School of Materials Science and Engineering, Shanghai Jiao Tong University Shanghai China; h Innovation Center for Future Materials, Zhangjiang Institute for Advanced Study, Shanghai Jiao Tong University Shanghai China; i Department of Chemical Engineering & Applied Chemistry, University of Toronto Canada; j Vector Institute for Artificial Intelligence Toronto Canada; k Canadian Institute for Advanced Research (CIFAR) Toronto Canada; l Acceleration Consortium, University of Toronto Toronto Canada

## Abstract

The search for new materials can be laborious and expensive. Given the challenges that mankind faces today concerning the climate change crisis, the need to accelerate materials discovery for applications like water-splitting could be very relevant for a renewable economy. In this work, we introduce a computational framework to predict the activity of oxygen evolution reaction (OER) catalysts, in order to accelerate the discovery of materials that can facilitate water splitting. We use this framework to screen 6155 ternary-phase spinel oxides and have isolated 33 candidates which are predicted to have potentially high OER activity. We have also trained a machine learning model to predict the binding energies of the *O, *OH and *OOH intermediates calculated within this workflow to gain a deeper understanding of the relationship between electronic structure descriptors and OER activity. Out of the 33 candidates predicted to have high OER activity, we have synthesized three compounds and characterized them using linear sweep voltammetry to gauge their performance in OER. From these three catalyst materials, we have identified a new material, Co_2.5_Ga_0.5_O_4_, that is competitive with benchmark OER catalysts in the literature with a low overpotential of 220 mV at 10 mA cm^−2^ and a Tafel slope at 56.0 mV dec^−1^. Given the vast size of chemical space as well as the success of this technique to date, we believe that further application of this computational framework based on the high-throughput virtual screening of materials can lead to the discovery of additional novel, high-performing OER catalysts.

## Abstract 2

The search for new materials can be laborious and expensive. With computational inverse design, material properties can be predicted before synthesis, thus accelerating materials discovery.

## Graphical abstract

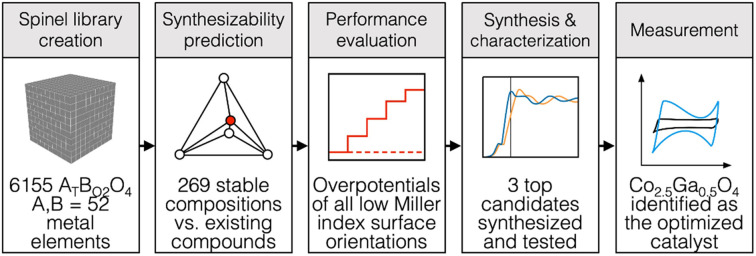

## Introduction

While hydrogen does represent a promising form of green energy storage, the sluggish kinetics of the water splitting reaction limits the efficiency and the practical implementation of electrolytic water splitting and green hydrogen production on an industrial scale.^1–3^ The current state-of-the-art materials for OER catalysis often contain materials like IrO_2_ and RuO_2_.^2,3^ RuO_2_ for example, typically has overpotentials between 250-350 mV at 10 mA cm^−2^ of current density and Tafel slopes between 50-70 mV dec^−1^ in basic conditions of 1 M KOH.^35,46,51^ However, IrO_2_ and RuO_2_ are rare and expensive, necessitating the development of cheaper catalysts that are comparable to them on both metrics.

**Fig. 1:**
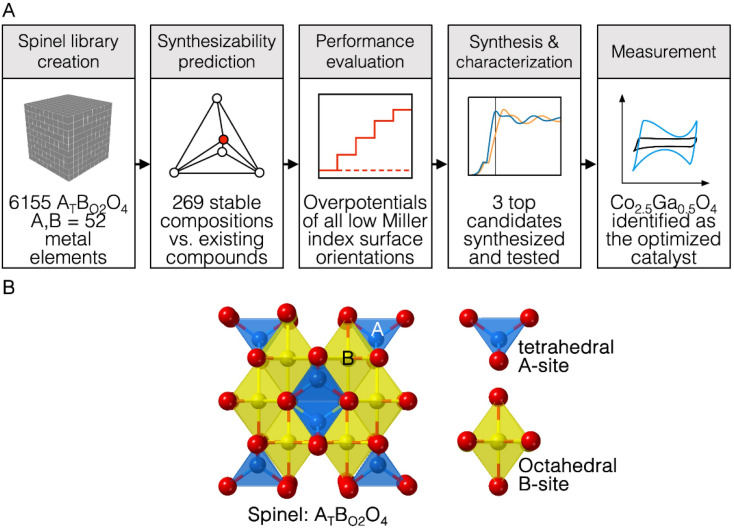
(A) Scheme describing the computational workflow to identify new catalysts for OER. In this workflow, Co_2.5_Ga_0.5_O_4_ was discovered as a highly-performing spinel oxide catalyst. (B) Diagram describing the structure of a spinel oxide AB_2_O_4_, where A is tetrahedrally coordinated and B is octahedrally coordinated.

Spinels can potentially replace IrO_2_ and RuO_2_ with earth-abundant metal oxide catalysts capable of catalysing OER.^2–5^ The crystal structure of spinel oxides is made up of oxygen anions arranged within a cubic close-packed sublattice while metallic cations are positioned within the tetrahedral and octahedral interstitial sites between the anions.^6,7^ The basic composition of a ternary-phase spinel oxide is A_*x*_B_3−*x*_O_4_, where A and B are two different metals.^6,7^ In this structure, the distribution of both metals across both coordination geometries can be represented by the formula (A_*x*−*ε*_B_1−*x*+*ε*_)_*T*_d__(A_*ε*_B_2−*ε*_)_*O*_h__ where *T*_d_ refers to tetrahedrally coordinated cations, *O*_h_ refers to octahedrally coordinated cations and *ε* refers to the inversion parameter (*ε* = 0 – *x*)^7^ (Fig. 1B). If *ε* equals 0, the structure is a normal spinel, and if *ε* equals *x*, the structure is an inverse spinel.^7^ The presence of transition metals in both the AO_4_ and BO_6_ structural units likely allows both of them to contribute to OER activity. This could potentially increase the structural diversity and tunability of spinel oxide catalysts for OER catalysis, making them an interesting system to study.

In recent years, spinels have proven to be a rich source of earth abundant metal oxide catalysts capable of catalyzing OER efficiently.^5^ Most spinel oxides reported for OER tend to be Fe-based or Co-based, with some examples of highly active spinel oxides being NiFe_2_O_4_, CoFe_2_O_4_ and CuCo_2_O_4_.^5^ For example, Liu and coworkers fabricated NiFe_2_O_4_ nanospindles on an Fe_3_Ni foam substrate that exhibited an overpotential of 262 mV at a current density of 10 mA cm^−2^, as well as a Tafel slope of 39.5 mV dec^−1^ in 1 M KOH.^8^ Lu and coworkers synthesized CoFe_2_O_4_ nanoparticles on carbon nanorods that had an overpotential of 240 mV at 10 mA cm^−2^ as well as a Tafel slope of 45 mV dec^−1^ in 1 M KOH.^9^ Yadav and coworkers synthesized 3D CuCo_2_O_4_ nanoflowers on a carbon cloth substrate that had an overpotential of 288 mV at 10 mA cm^−2^ and a Tafel slope of 64.2 mV dec^−1^ in 1 M KOH.^10^ The efficacy of these catalysts demonstrate that spinels could be a promising source of new materials for OER catalysis. However, while considerable work has been done so far to discover new spinel oxide catalysts for OER, the entire possible chemical space of spinels has yet to be explored. There could potentially be more efficient spinel oxide catalyst compositions that haven't been discovered yet.

It is not feasible to explore the entire chemical space experimentally, due to the immense number of permutations possible with each element in the periodic table. According to one estimate by Walsh and coworkers, the number of possible ternary phase inorganic materials that can be synthesized out of 103 elements in the periodic table is greater than 32 million, even after constraining this space by imposing charge neutrality and electronegativity rules.^11^ However, it is possible to explore a greater fraction of this space computationally, in order to narrow down possible candidates before synthesizing them in a lab. This approach towards materials discovery has had a significant impact in fields as diverse as catalysis,^12,13^ Li-ion batteries,^14,15^ thermoelectrics,^16,17^ organic light-emitting diodes,^18^ and transparent conducting oxides.^19^ Within the field of catalysis, there have been high-throughput computational studies that have aimed to discover new OER catalysts. For example, Xu and coworkers utilized a bandcenter descriptor to screen 3d spinel oxides for OER activity.^13^ Ulissi and coworkers screened 2600 equimolar Ir-containing bimetallic oxides for acid-stable OER catalysts and identified 14 possible candidates predicted to be stable under acidic conditions.^12^ Nørskov and coworkers screened 47 814 nonbinary metal oxides in the Materials Project and identified 68 possible acid-stable OER catalysts.^20^ In each of these studies, computational screening was used to predict the stability or activity of OER catalysts. However, to the best of our knowledge, no study has successfully utilized a high-throughput computational screen to discover novel, highly-active OER catalysts that have been experimentally demonstrated to compete with current benchmark catalysts reported in the literature. In this paper, we discuss a high-throughput computational workflow that enabled us to achieve this goal.

In this study, we built a computational workflow to screen for new highly active spinel oxides for OER in basic pH conditions. A computational database of ternary-phase spinel oxide materials comprised of 52 elements in the periodic table is first constructed. These elements include alkaline, alkaline-earth, transition (with the exception of artificial Tc), post-transition and some lanthanide metal elements (La, Ce, Nd, Gd and Lu) and are circled in Fig. S1.† Every possible combination of elements that could be constructed from this list was explored, for an overall spinel system A_*x*_B_3−*x*_O_4_ where A and B are two different metallic elements. For each system A_*x*_B_3−*x*_O_4_, three different values of *x* (*x* = 0.5, *x* = 1, *x* = 1.5) are chosen in order to screen for three unique compositions A_0.5_B_2.5_O_4_, AB_2_O_4_ and A_1.5_B_1.5_O_4_. Both normal spinel (*ε* = 0) and inverse spinel (*ε* = *x*) structures for each specific composition were explored. Once this database was created, the thermodynamic stability of these materials was assessed to isolate materials that are likely to exist in nature. Following that, the theoretical overpotential of OER on the surface of these materials was computed. We used the results of these computations to create a machine learning model to predict OER activity using electronic structure descriptors of bulk metal, bulk oxygen, surface metal, surface oxygen and adsorbate oxygen atoms as features. This allowed us to further probe the relationship between electronic structure descriptors and OER activity. The trends observed in the results of this high-throughput computational screen were applied to synthesize three catalysts, Co_2.5_Ga_0.5_O_4_, Co_1.5_Ga_1.5_O_4_ and Co_1.5_Al_1.5_O_4_ and characterize them for OER activity. We discovered that Co_2.5_Ga_0.5_O_4_ had an overpotential of 220 mV at a current density of 10 mA cm^−2^ and a Tafel slope of 56.0 mV dec^−1^, making it a new highly active OER catalyst successfully discovered using this novel computational workflow. The success of this workflow at predicting a real catalyst highly active for OER implies that it can be adapted to discover new highly active OER materials belonging to other classes of materials as well.

## High throughput computational workflow

A computational database of 6155 ternary-phase spinel oxides was first created from 52 transition, post-transition, alkaline, alkaline earth and lanthanide metal elements. For each combination of metal elements, three different ratios of metals were explored: A_0.5_B_2.5_O_4_, AB_2_O_4_ and A_1.5_B_1.5_O_4_. Both normal and inverse spinels of each permutation of materials were also considered, leading to six different spinel structures: (A_0.5_B_0.5_)_*T*_d__(B_2_)_*O*_h__O_4_, (B)_*T*_d__(A_0.5_B_1.5_)_*O*_h__O_4_, (A)_*T*_d__(B_2_)_*O*_h__O_4_, (B)_*T*_d__(AB)_*O*_h__O_4_, (A)_*T*_d__(A_0.5_B_1.5_)_*O*_h__O_4_ and (B)_*T*_d__(B_0.5_A_1.5_)_*O*_h__O_4_. The spinel oxide Fe_3_O_4_ belonging to the *Fd*3*m* spacegroup was used as a prototype structure for every single combination of elements except those utilizing Mn; for spinel oxides containing Mn in the octahedral positions, Mn_3_O_4_ was used as a prototype structure instead in order to account for the Jahn–Teller distortion of octahedrally coordinated Mn^3+^ ions.^21^ DFT calculations were subsequently performed to relax the structure of each compound and obtain its ground state energy.

In the next step of the high-throughput workflow, the thermodynamic stability of each composition was assessed. First, the energies of the normal and the inverse spinel structures of each composition were compared to determine the most probable ground-state configuration of each composition, with the more stable one being chosen for the next step. Then, we constructed a convex hull based on the bulk energies of all known materials encompassing the phase space of the elements that constitute each spinel oxide composition. The Open Quantum Materials Database was used to construct the phase space of the elements that constitute each compound.^22,23^ The thermodynamic stability of each composition was subsequently determined by calculating the distance of the bulk energy of each composition from this convex hull. The total energy of each compound was compared to a linear combination of possible decomposition products that lie on the convex hull of the phase space in order to calculate this distance from the convex hull.^15,24^ If the difference between the energies was within 0.030 eV per atom (30 meV per atom), the compound would be considered thermodynamically stable.^25^

**Fig. 2:**
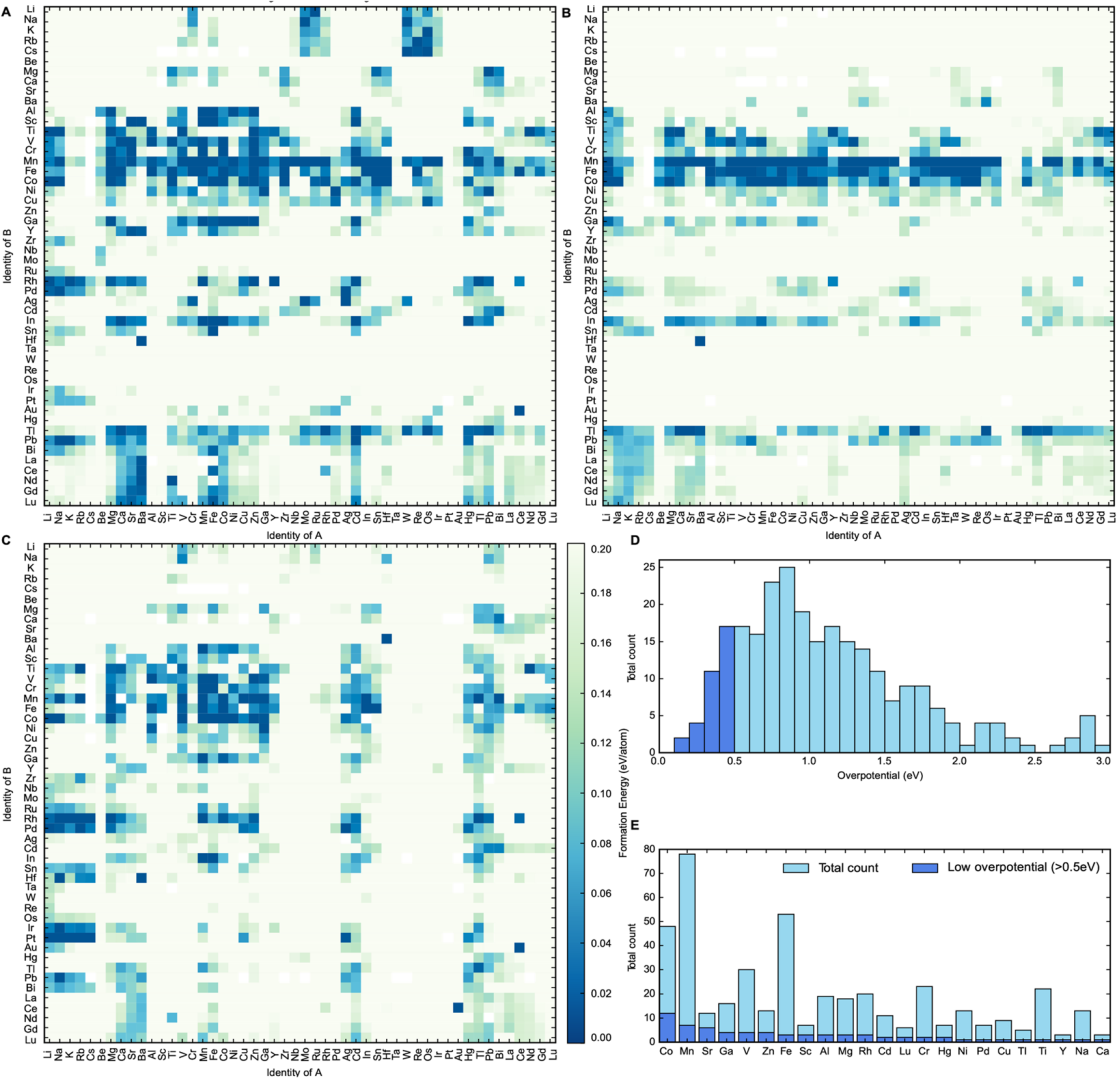
Heat map outlining the stability of compositions (A) AB_2_O_4_, (B) A_0.5_B_2.5_O_4_ and (C) A_1.5_B_1.5_O_4_. Stable spinels in this study are considered to have formation energies below 0.03 eV per atom with respect to the convex hull. (D) Frequency plot of all thermodynamically stable materials based on the calculated theoretical overpotential. Materials deemed to have a low theoretical overpotential (less than 0.5 eV) marked in dark blue. (E) Frequency of each metallic element amongst thermodynamically stable spinels for which theoretical overpotentials for OER were also calculated. Elements making up low overpotential compounds (less than 0.5 eV) are also labelled in dark blue.

The heat maps in Fig. 2A–C show the formation energies of the different spinel oxide compositions. The higher density of formation energies below 0.030 eV per atom demonstrates that the AB_2_O_4_ spinel composition is generally the most stable composition type, followed by A_0.5_B_2.5_O_4_ and then A_1.5_B_1.5_O_4_. This is probably because in the latter two compositions, at least one of the cations should occupy both tetrahedral and octahedral sites in the spinel oxide structure. This cation should be able to assume both +2 and +3 oxidation states and should not have any strong preference for tetrahedral or octahedral coordination geometry. This constraint would limit the number of possible stable compositions for both A_0.5_B_2.5_O_4_ and A_1.5_B_1.5_O_4_ compositions relative to AB_2_O_4_. It can be observed that spinel oxides containing 3d metal cations are typically more stable than spinel oxides containing 4d and 5d metal cations. Mn, Co and Fe-containing spinels, in particular, are more likely than other metal cations to have formation energies that are within 0.030 eV of their convex hulls. This makes Mn, Co and Fe-containing spinels more likely to exist in nature.^25^ This observation can first be partly rationalized by the fact that Mn, Co and Fe are stable in the +2 and +3 oxidation states.^26^ Furthermore, according to Kocevski and coworkers, Mn, Co and Fe ions do not have a strong preference for either tetrahedral or octahedral sites in spinels and are frequently seen in either of them.^27^ Therefore, Co, Mn and Fe containing spinel structures have a greater amount of flexibility in accommodating a wider variety of other cations as a result, making them more ubiquitous than other elements; an observation that was further corroborated by the calculations performed in this work.^27^ Spinels containing metals of higher oxidation states can be stabilized through the engineering of oxygen vacancies into the structure.^6^ This study, however, did not consider defects such as oxygen vacancies in order to keep computational costs reasonable. After this step had been performed, 290 materials were identified as being potentially thermodynamically stable. They were selected for the final step of this high-throughput computational screen, where their OER activity was estimated.

In the final step of this workflow, the catalytic activity of each of these 290 spinel oxide materials is predicted by calculating the theoretical overpotential using the computational hydrogen electrode method proposed by Nørskov and coworkers.^28^ The computational hydrogen electrode states that the chemical potential of the proton and electron pairs can be described as half of the chemical potential of the H_2_ molecule since these species are in chemical equilibrium at 0 V *vs.* the reversible hydrogen electrode (RHE).^28^ We can use this model to estimate the Gibbs free energies of the first two reaction steps of the adsorbate evolution mechanism (AEM) by calculating the binding energies of the *O, *OH and *OOH intermediates of OER. To calculate these energies, we created slabs of each material, relaxed the surface of the slabs, and then added the relaxed *O, *OH and *OOH molecules on top of the metal sites of the slab surface. All possible slab surfaces with a maximum absolute Miller index of one were first created using Pymatgen.^29–31^ Higher index surfaces were not included in this step to keep the computational cost reasonable. The surface energies of all these slabs were calculated and the slab with the lowest surface energy was identified. Next, *O, *OH and *OOH molecules were fixed onto the surface of the slab with the lowest surface energy. Each of these slabs has a 50% monolayer coverage of *O, *OH and *OOH intermediates respectively. This assumption was made in order to limit computational cost that could come with considering all the different permutations of intermediates possible. We note that surface coverages can change depending on the applied potential, and that the potential window of the stability of surface coverage should be taken into account in future studies where the predicted overpotential of catalysts is computationally assessed. The adsorption energy of each of these intermediates was calculated and used to determine the theoretical overpotential of OER on the surface of each material.

The distribution of the calculated theoretical overpotentials of the compounds is plotted in Fig. 2D. Out of the 290 materials for which these theoretical overpotential calculations were successfully performed, 33 compounds show potential to be useful OER catalysts with theoretical overpotentials below 0.5 eV. Spinel oxides like CuCo_2_O_4_ and CoFe_2_O_4_ that are known in the literature to be highly active for OER were also present amongst these 33 compounds.^5,9,10^ This further confirms the efficacy of this screen at discovering real materials that catalyze OER efficiently. To better understand the impact of elemental composition on catalytic activity, a frequency plot of the elements in all calculated compounds is plotted with respect to theoretical overpotential in Fig. 2E. It seems that Cobalt forms the largest number of catalytically active spinel oxides for OER, since they have the greatest number of low overpotential (<0.5 V) compounds compared to others. This insight corroborates existing trends in the literature on the use of spinel oxides for OER since most spinels reported for OER are either Co or Fe-based.^5^

We then use the results of these OER calculations to train a machine learning model to predict the binding energies of the *O, *OH and *OOH intermediates of OER.

## Predicting *O, *OH and *OOH binding energies based on electronic structure descriptors

**Fig. 3:**
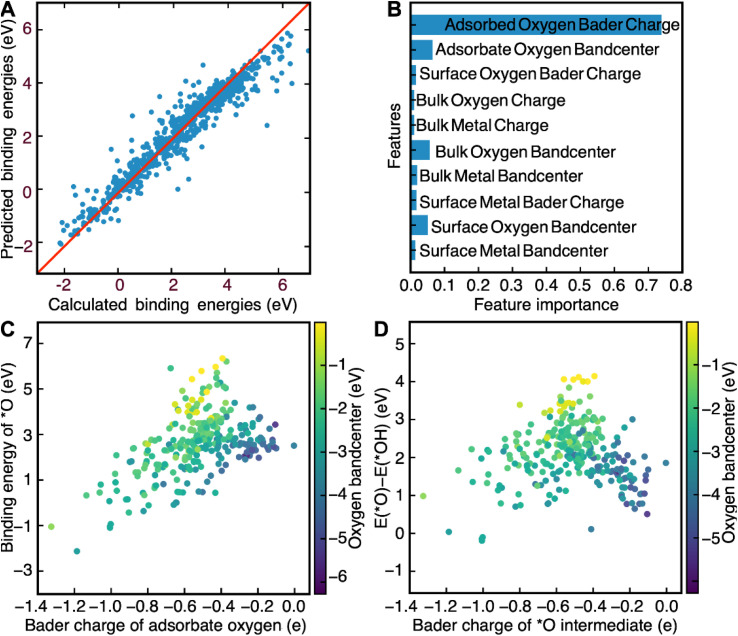
(A) A graph created to illustrate the efficacy of the random forest model by comparing calculated binding energies of all the *O, *OH and *OOH intermediates with predicted binding energies on the test set. The model has an *R*^2^ score of 0.83 for the training set and 0.78 for the test set. It also has a median absolute error of 0.16 eV for the training set and 0.21 eV for the test set. (B) A bar graph illustrating the relative importance of all the features used to predict the binding energies of *O, *OH and *OOH. The Bader charge of the adsorbed oxygen was plotted against the (C) binding energy of *O and (D) the difference in energy between O* and *OH, with the colorbar representing the bandcenter of the O2p band of adsorbate oxygen.

Now that we have demonstrated that the success of this computational workflow at discovering new catalysts, we have decided to use the database created by this workflow to train a machine learning model to predict the binding energies of *O, *OH and *OOH intermediates. The objective of this task is to use this model to identify patterns, based on DFT-calculated electronic structure descriptors, that could aid with the swift screening of OER catalysts. Electronic structure descriptors have been successfully used in previous studies as a computationally inexpensive means of predicting OER activity.^69–72^ The bulk 3d band center in metallic catalysts and the bulk O2p bandcenter in perovskites have been shown to be predictive of OER activity in previous studies.^66–68^ However, the main weakness of solely using bulk descriptors is the likelihood that surface effects end up getting ignored. Stoerzinger and coworkers demonstrate this reality when they showed that OER activity on the surface of RuO_2_(100) and RuO_2_(101) was higher than their (110) and (111) counterparts in basic conditions due to the higher concentration of coordinatively unsaturated sites.^73^ Any comprehensive assessment of the link between OER activity and electronic structure descriptors would have to include electronic structure descriptors associated with both bulk and surface atoms as well. Inspired by a recent study by Lunger and coworkers which concluded that the Bader charge and the O2p bandcenter of surface oxygen was predictive of *O, *OH and *OOH binding energies on the surface of perovskite slabs, we decided to use the bandcenters and Bader charges of the atoms in each slab as features for our model.^71^ We first calculated the O2p bandcenters of the surface oxygen, bulk oxygen and adsorbate oxygen atoms, the M3d bandcenters of the surface metal and bulk metal atoms and the Bader charges of the surface oxygen, bulk oxygen, adsorbate oxygen, surface metal and bulk metal atoms. These descriptors were then used as training data for a random-forest regression model. We used a random-forest model since they are known to be simple to use, accurate, and capable of dealing with small sample sizes and high-dimensional feature spaces.^75^ In addition, since random forests are also interpretable, we can also look at the relative importance of the input features to find electronic structure descriptors that can correlate well with predicted OER activity.^75^ This model was able to predict the binding energies of *O, *OH and *OOH intermediates quite accurately, with an *R*^2^ score of 0.83 for the training set and 0.78 for the test set (Fig. 3A). We also found that the outliers of our model, defined as a prediction which differs from the calculated value by more than 1 eV, were far more likely to contain novel elements like Nb, Pb, Cd, and Ir. The failure of our model to predict the theoretical overpotentials of these structures can be attributed to the fact that these materials were underrepresented in our dataset, since spinel oxides fabricated using these elements were far more likely to be unstable and were therefore filtered out by the convex hull stability screen. Such outliers, thankfully, are rare and can only be attributed to 4% of the calculations in our screen, further demonstrating the efficacy of our model at predicting OER binding energies.

The relative importance of each feature for the prediction of binding energies was then extracted from the random forest model. The Bader charge of adsorbate oxygen was the most important feature of all of them, with the O2p bandcenter of the adsorbate oxygen atom coming at a distant second (Fig. 3B). The significance of electronic structure features associated with adsorbate oxygen for OER is corroborated by previous work by Nørskov and coworkers, which demonstrated a link between the O2p bandcenter of adsorbate oxygen and the calculated theoretical overpotential.^69^ We also decided to use LASSO to predict the binding energies of the OER intermediates, in order to determine how universal the features we identified were with respect to their relationship with the binding energies of OER (Fig. S14†). Just like with the random-forest model, the Bader charge of adsorbed oxygen was determined to be the most important feature. The second most important feature in the LASSO model, however, was deemed to be the Bader charge of surface oxygen. The LASSO model performed poorly at predicting binding energies compared to the random-forest model; with an *R*^2^ value of 0.65 for the training set, an *R*^2^ value of 0.72 for the test set and a median absolute error of 0.56 eV for both the training and the test sets. As such, we decided to use the results of the random-forest model for further analysis instead.

We then further examine the relationship between the two most important features identified by our random-forest model, the Bader charge and the O2p bandcenter of adsorbate oxygen, and the binding energies of *O, *OH and *OOH. We plot the relationship between the binding energies of *O and *OH and demonstrate that any increases in the Bader charge of adsorbate oxygen can be associated with an increase in the binding energies of the *O, *OH and *OOH intermediates. Furthermore, increases in the distance between the O2p bandcenter and the Fermi level are associated with decreases in the binding energies of *O (Fig. 3C) and *OH (Fig. S13†). We then plot Δ*E*_O_–Δ*E*_OH_ against both the oxygen bandcenter and the Bader charge of the adsorbate oxygen in the *O intermediate (Fig. 3D). We see that the relationship between Δ*E*_O_–Δ*E*_OH_ and the Bader charge of oxygen resembles that of a volcano plot; Δ*E*_O_–Δ*E*_OH_ increases when going from a Bader charge of −1.4e^−^ to −0.5e^−^ before decreasing against from −0.5e^−^ to −0.1e^−^. Δ*E*_O_–Δ*E*_OH_ decreases as the O-2p bandcenter moves further away from the Fermi level, a relationship that can be corroborated by Nørskov and coworkers.^69^ Since the optimal Δ*E*_O_–Δ*E*_OH_ would be between 1.5 and 1.7 eV, the ideal catalyst is likely to have an oxygen Bader charge between −0.3e^−^ and −0.6e^−^ and an O-2p bandcenter between 2 eV and 4 eV below the Fermi level. Screening for catalysts based on both descriptors is likely to lead to the identification of more highly active catalysts for OER.

## Synthesis and characterization of compounds

**Fig. 4:**
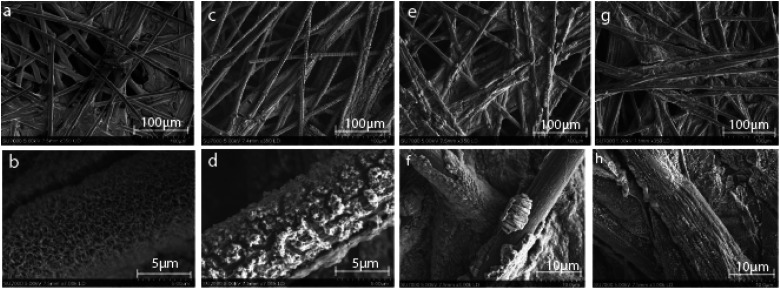
SEM images of all four catalysts with two different resolutions. The four images on the top show zoomed out images of catalysts presumed to be (a) Co_3_O_4_, (c) Co_1.5_Al_1.5_O_4_, (e) Co_2.5_Ga_0.5_O_4_ and (g) Co_1.5_Ga_1.5_O_4_. The images at the bottom show zoomed in images of the same catalysts thought to be (b) Co_3_O_4_, (d) Co_1.5_Al_1.5_O_4_, (f) Co_2.5_Ga_0.5_O_4_ and (h) Co_1.5_Ga_1.5_O_4_.

Out of the 33 possible candidates predicted to have theoretical overpotentials below 0.5 V, three Co-based spinel oxides, Co_1.5_Al_1.5_O_4_, Co_2.5_Ga_0.5_O_4_, Co_1.5_Ga_1.5_O_4_, were selected for synthesis and experimental characterization based on their novelty. They were synthesized *via* electrodeposition onto a carbon paper substrate. In order to obtain compounds that have a similar composition to these predicted materials, stoichiometric mixtures of the salts Co(NO_3_)_2_, Ga(NO_3_)_3_ and Al(OH)(C_2_H_3_O_2_)_2_ were dissolved in the electrolyte solution used for electrodeposition in all three cases. Co_3_O_4_ was also synthesized for benchmarking purposes since it is well-known to be active for OER.^49^ After synthesizing these materials, their morphologies and chemical compositions were characterized with SEM and energy dispersive X-ray spectroscopy (EDS) mapping. The SEM images of all four catalysts (Fig. 4) reveal the presence of an amorphous material evenly deposited on the surface of the carbon substrate. EDS maps (Fig. S2–S5†) of Co_3_O_4_ and Co–Al oxide indicate that all the constituent elements of both materials are uniformly distributed. In contrast, the elements in the Co–Ga oxide samples show greater heterogeneity in their distribution over the surface of the material. This seems to indicate the presence of another phase.

**Fig. 5:**
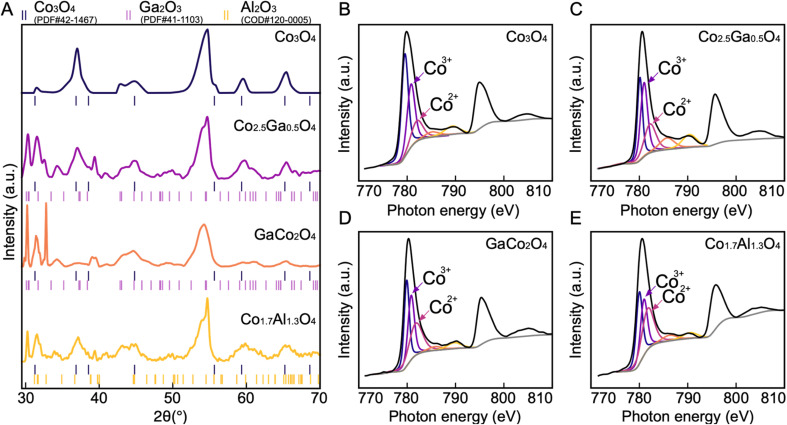
XRD and XPS spectra of all four compounds. (A) XRD spectra of all four catalysts. The spinel phase is present in all four catalysts, and in Co_2.5_Ga_0.5_O_4_, Co_1.5_Ga_1.5_O_4_ and Co_1.5_Al_1.5_O_4_, the phases of Ga_2_O_3_ and Al_2_O_3_ are present as well. Co 2p XPS spectra of materials presume to be (B) Co_3_O_4_ (C) Co_2.5_Ga_0.5_O_4_ (D) Co_1.5_Ga_1.5_O_4_ (E) Co_1.5_Al_1.5_O_4_. In order to determine the composition, the Co 2p^3/2^ peak was deconvoluted. The peaks corresponding to Co^2+^ and Co^3+^ are labelled in each XPS diagram.

To further investigate the structure of the synthesized catalysts, all four materials were characterized by XRD (Fig. 5A). The major peaks corresponding to the spinel phase are found at 31.3°, 36.9°, 44.8°, 59.4° and 65.2°, which are the characteristic peaks of Co_3_O_4_.^32^ The peaks at 44.8° and 55.0° found in all four spectra can be attributed to the carbon paper substrate (Fig. S11†). The presence of the spinel peaks in all four spectra demonstrates that spinel oxides have been successfully synthesized in all four cases. However, in addition to the peaks corresponding to the spinel oxide and the carbon substrate phases, new peaks corresponding to a different phase also appear in the Co–Ga and Co–Al oxide spectra. These can be attributed to β-Ga_2_O_3_ in Co–Ga oxide spectra and θ-Al_2_O_3_ in the Co–Al oxide spectrum.^32,33^ Since neither Ga_2_O_3_ nor Al_2_O_3_ are redox-active, they are unlikely to make any significant contribution to OER activity.^34,35^

The composition of the synthesized materials was characterized with XPS (Fig. S6–S9†). The high-resolution Co2p spectra of each of these materials (Fig. 5B–E) show evidence of spin–orbit splitting into 2p_3/2_ and 2p_1/2_ components at ∼780 eV and ∼795 eV respectively, with shake-up satellites for each of these components present at ∼790 eV and ∼805 eV.^36^ Each of these spectra resembles the Co2p spectra of Co_3_O_4_, further corroborating the results of the XRD that have demonstrated the successful synthesis of cobalt spinel oxide.^36^ In order to determine the stoichiometry of each of these catalysts, the Co2p^2/3^ spectra were deconvoluted based on the Co2p^2/3^ peak fitting parameters by Biesinger *et al.* and the ratio between the areas of the peaks associated with the Co^2+^ and Co^3+^oxidation states were calculated.^36,37^ Since both Ga and Al are not stable in the +2 oxidation state, they are both likely to displace Co^3+^ if successfully incorporated into the spinel oxide structure. Thus, comparing the Co^2+^ and Co^3+^ ratio will help determine the stoichiometry. The ratios of the peak areas of Co^2+^ to Co^3+^ are 1 : 2, 1 : 0.7, 1 : 1.5 and 1 : 1 in the Co_3_O_4_, Co–Al, and the two Co–Ga catalysts respectively. This indicates that the exact structural formulae of the catalysts are Co_3_O_4_, Co_1.7_Al_1.3_O_4_, Co_2.5_Ga_0.5_O_4_ and GaCo_2_O_4_.

## Catalytic characterization

**Fig. 6:**
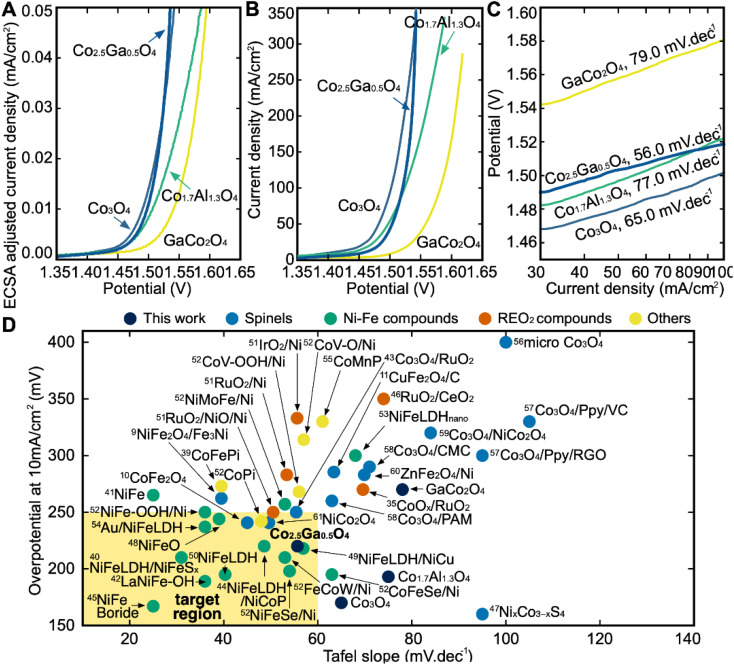
(A) ECSA-adjusted *iR*-corrected LSV curves of all catalysts outlining the current density at different potentials (*vs.* RHE). Linear sweep voltammetry (LSV) measurements are performed in 1 M KOH solution at pH 14. (B) *iR*-corrected LSV curved of all catalysts. (C) Tafel Slopes of all catalysts shown on this semilog plot. Co_2.5_Ga_0.5_O_4_ has the lowest Tafel slope of all the catalysts. (D) Benchmarking catalysts in this study with other state-of-the-art catalysts reported in the literature. Each experiment in our plot was done in 1 M KOH solution. A more detailed breakdown of each reference catalyst can be seen in Table S5.†

After the synthesis and structural characterization of these catalysts, they were electrochemically characterized for OER activity by linear sweep voltammetry (LSV) in a 1 M KOH solution (Fig. 6B). The overpotential in this report is defined as the *iR*-corrected potential at 10 mA cm^2^_geo_^−1^ minus 1.23 V, where geo represents the geometric surface area. At 10 mA cm^2^_geo_^−1^ of current density, Co_3_O_4_ has the lowest overpotential of all the catalysts at 170 mV, followed by Co_1.7_Al_1.3_O_4_ at 193 mV, Co_2.5_Ga_0.5_O_4_ at 220 mV and finally GaCo_2_O_4_ at 270 mV. However, while Co_2.5_Ga_0.5_O_4_ has a higher overpotential than either Co_3_O_4_ or Co_1.7_Al_1.3_O_4_, it also has a much lower Tafel slope at 56.0 mV dec^−1^ than either catalyst (Fig. 6C). This allows Co_2.5_Ga_0.5_O_4_ to start outcompeting the rest of the catalysts at around 1.55 V_RHE_.

The LSV plot of each catalyst was further corrected using the estimated electrochemically active surface area (ECSA) of each catalyst in order to determine the intrinsic activity of each material for OER (Fig. S10†). Co_3_O_4_ has the highest ECSA out of all four catalysts, followed by Co_1.7_Al_1.3_O_4_, Co_2.5_Ga_0.5_O_4_ and then GaCo_2_O_4_. After adjusting for the ECSA of all catalysts (Fig. 6A), Co_2.5_Ga_0.5_O_4_ starts outcompeting Co_3_O_4_ even earlier, at about 1.52 V_RHE_.

## Benchmarking catalysts against state-of-the-art materials

The catalysts explored in this report were then compared to 38 other benchmark catalysts in the literature in order to assess their performance (Fig. 6D).^8–10,38–61^ The catalysts explored in this report were compared to four families of catalysts: Non-spinel Co catalysts (marked as Others), spinel oxide catalysts, Ni–Fe catalysts and Ru/Ir-based catalysts. The ideal catalyst would be one with a lower Tafel slope and a lower overpotential than the state-of-the-art catalysts in the literature that currently exist. While Co_1.7_Al_1.3_O_4_ has a low overpotential at a current density of 10 mA cm^−2^, its high Tafel slope compared to the benchmark catalysts examined makes it uncompetitive. The performance of the GaCo_2_O_4_ catalyst with respect to its overpotential and Tafel slope was also uncompetitive compared to the benchmark catalysts. On the other hand, Co_2.5_Ga_0.5_O_4_ is as competitive as some of the better benchmark materials within the Ni–Fe family of catalysts on both the overpotential and Tafel slope metrics. It has also outperformed all the Co, spinel oxide and Ru catalysts examined in this report, with respect to both its overpotential and its Tafel slope. The high performance of Co_2.5_Ga_0.5_O_4_ demonstrates the efficacy of this high-throughput computational workflow at discovering promising OER catalysts.

## Conclusions

In this work, a high-throughput computational framework was developed in order to screen for novel low-cost materials for OER in basic conditions. Out of the 6155 binary spinel oxides that were investigated for this work, 33 were predicted to have low theoretical overpotentials below 0.5 eV. This made them ideal candidates for further study through experiment. Based on an analysis of the results of the overpotential screen, Ga and Al-doped Co_3_O_4_ were investigated using LSV to determine their OER activity. The data indicated that Ga_0.5_Co_2.5_O_4_ was highly active for OER, surpassing many other state-of-the-art catalysts that have been reported in the literature. This catalyst is, to the best of our knowledge, novel and has not been investigated for OER before. These results further demonstrate the strength of this computational framework for facilitating the discovery of novel materials for OER.

## Methods

### DFT calculation details

DFT calculations were performed to optimize the structure of each spinel oxide in this database and calculate their energies. Every DFT calculation in this study was performed with the Vienna *ab initio* Simulation Package (VASP). The Projector-Augmented Wave (PAW) method was used to model the core electrons.^62^ The Perdew–Burke–Ernzerhof functional which utilizes the generalized gradient approximation approach was used to describe the exchange-correlation effects.^63^ For spinel oxides containing 3d metal elements with the exception of Zn, spin-polarized DFT calculations were performed; for all other materials non spin-polarized calculations were performed instead. The Hubbard *U* correction was employed for materials containing 3d transition metals; the exact values used in this study are described in ESI Table S4.† The energy cut-off used in these calculations was 520 eV. Each and every single one of these parameters was optimized to ensure that the right balance between computational cost and accuracy was achieved.

In the first step of this screen, three structural optimizations were performed on the bulk structure of each spinel oxide within this constructed dataset before the energy of each structure was determined. A gamma-centered *k*-point mesh with a density of 8000 *k*-points/number of unit cell atoms was generated for each material using Pymatgen.^29^ Both the shape of the unit cell and the positions of the atoms were allowed to fluctuate until the energy convergence criterion of 10^−5^ eV between each self-consistent field iteration step and the force convergence criterion of 0.01 eV Å^−1^ were met. Once the relaxation steps had been concluded, the shape of the unit cell and the positions of the atoms were fixed in order to evaluate the energy of each structure. The Brillouin zone was integrated using the tetrahedron method with Blöchl corrections for this energy calculation.^64^

### Creating slabs and calculating the binding energies of the *O, *OH and *OOH intermediates

When creating all slabs using Pymatgen, the width of each slab was set at 6 Å and the vacuum space was set at 20 Å. The energies of each slab were then calculated using DFT. Once the slab energies had been calculated, the surface energy of each slab was calculated using the following formula:^31^
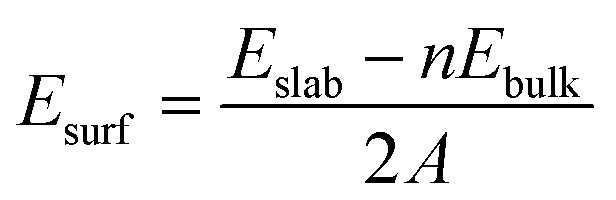
where *E*_surf_ is the surface energy, *E*_slab_ is the calculated energy of the slab, *E*_bulk_ is the energy (per atom) of the bulk structure, *n* is the number of atoms in a unit cell and *A* is the surface area of the slab. The slab with the lowest surface energy was then isolated and utilized for the final step of this screen.

VASP was used in the DFT calculations of slab energies. The top two layers of atoms in each slab were allowed to relax for two steps while the bottom layers were frozen. A gamma-centered *k*-point mesh with a density of 1000 *k*-points/number of unit cell atoms was generated for each material using Pymatgen.^29^ In each structural optimization step, the atoms in the top two layers were allowed to relax until the energy convergence criterion of 10^−5^ eV and the force convergence criterion of 0.1 eV Å^−1^ were met. Once the relaxation steps had been concluded, the positions of all atoms in each slab was fixed in order to evaluate the energy of the slab, where the tetrahedron method with Blöchl corrections was used to integrate the Brillouin zone.^64^ The energy cutoff used in these slab calculations was 400 eV. The slab surface with the lowest surface energy was used to calculate the binding energies of the *O, *OH and *OOH intermediates.

The binding energies of the *O, *OH and *OOH intermediates were then calculated by adsorbing each molecule on top of the metal sites on the relaxed surfaces of the slab with the lowest surface energy. Both A and B sites on the surface of the spinel catalysts were considered. The top two layers, along with the molecules, are allowed to relax for two steps while the bottom layers are kept fixed. The energies of each slab were calculated based on the same parameters used to calculate the surface energies in the previous step. The adsorption energies of the OER intermediates on each slab are subsequently calculated using the following equations:^28^Δ*G*_ads_ = Δ*E*_ads_ + Δ*E*_ZPE_ − *T*Δ*S*_ads_Δ*E*_ads_ = *E*_slab+OxHy_ − *E*_slab_ − *xE*_O_ − *yE*_H_*E*_O_ = *E*_H_2_O_ − *E*_H_2__
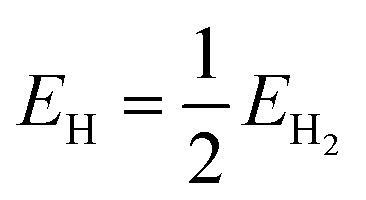
where Δ*G*_ads_ is the adsorption energy of the adsorbate (*O, *OH or *OOH), Δ*E*_ads_ is the electronic adsorption energy of the adsorbate, Δ*E*_ZPE_ is the zero-point vibrational energy difference between adsorbed and gaseous species, *T*Δ*S*_ads_ is the entropy difference between the gaseous and adsorbed species, *E*_slab_ is the energy of the clean slab, *E*_slab+OxHy_ is the energy of the slab with the adsorbate species on the surface, *E*_O_ is the energy of an oxygen atom, *E*_H_ is the energy of a hydrogen atom, *E*_H_2_O_ is the energy of a water molecule and *E*_H_2__ is the energy of a hydrogen molecule.

### Calculating the theoretical overpotential based on binding energies

Fig. S12† shows the different steps of the adsorbate evolution mechanism (AEM) proposed for water oxidation and the equations necessary to calculate each step of the mechanism. According to the computational hydrogen electrode model proposed by Nørskov and coworkers, steps 1 and 2 are typically the potential limiting steps of the AEM mechanism of OER.^28,65^ Therefore, in order to calculate the theoretical overpotential, Δ*G*_1_ and Δ*G*_2_ must be calculated based on the binding energies of the intermediates calculated in the previous section. 1.23 eV shall then be subtracted from the larger of the two energies in order to calculate the theoretical overpotential.^65^

### Creating the machine learning model

The random forest model was created using the Python package scikit-learn. Features such as the bandcenters of the oxygen and metal atoms were calculated, relative to the Fermi level, based on the formula below:
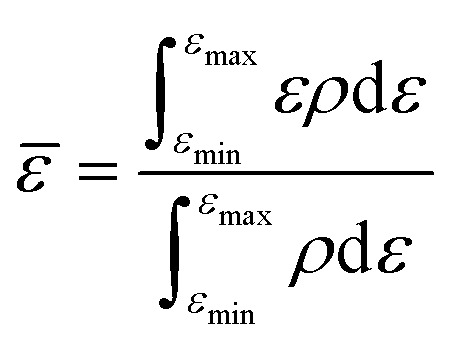
where *ε̄* refers to the bandcenter, *ε* refers to the energy level, *ρ* refers to the density of states at that energy level, *ε*_max_ refers to the maximum energy level within this range of integration and *ε*_min_ refers to the minimum energy level within this range of integration.

The Bader charge was calculated using a Bader charge analysis code provided by the Henkelman group.^74^ The average bandcenters and Bader charges of all atoms were used to train the random forest model. To train and test the models, the dataset was split using *train_test_split* from *scikit-learn*, saving 20% as test set. The optimal model hyperparameters were identified using a Bayesian optimization approach (*BayesSearchCV* from *scikit-optimize*, on *RandomForestRegressor* from *scikit-learn*) on the training set with a 5-fold cross validation. The hyperparameters of the model that were optimized were the number of trees in the random forest model, the maximum depth of each tree, the minimum number of samples required to split an internal node, the minimum number of samples required to be at a leaf node and the number of features to consider when looking for the best split. For comparison, LASSO was also tested to predict the binding energies of the OER intermediates (Fig. S14†). The same features used to train the random-forest model were used to train the LASSO model as well, and the hyperparameters were also optimized using Bayesian optimization. The dataset for the LASSO model was also split similarly to the random-forest model, with 20% of the dataset being saved for the test set. The hyperparameter tuned for the LASSO model is the alpha parameter that determines the magnitude of the L1 regularization term.

### Synthesis and characterization

#### Chemicals

All chemicals, including cobalt(ii) nitrate hexahydrate (Co(NO_3_)_2_·6H_2_O, 98%), gallium(iii) nitrate hydrate (Ga(NO_3_)_3_·*x*H_2_O, 99%), aluminum acetate (Al(OH)(C_2_H_3_O_2_)_2_, 99%), potassium hydroxide (KOH, 90%) were purchased from Sigma-Aldrich. Carbon paper (AvCarb MGL 190) substrates were purchased from the Fuel Cell Store. All chemicals were used without further purification. Millipore water (18.2 MΩ cm) was used in all experiments.

#### Synthesis of Co_3_O_4_ catalysts and metal cation doped Co_3_O_4_ catalysts on carbon paper substrates

These catalysts were synthesized using an electrodeposition method in a standard three electrode cell consisting of carbon paper as working electrode, carbon rod as a counter electrode and saturated calomel electrode(SCE) as reference electrode at the room temperature. Co(NO_3_)_2_·6H_2_O (0.1 M) was dissolved in water as the electrolyte. Carbon paper with the size of 0.5 cm × 0.5 cm was then immersed into the electrolyte for the electrodeposition of Co_3_O_4_-species. Electrodeposition was performed at a potential range of −1.0 to +0.2 V/SCE for 50 scans using an Autolab PGSTAT302N workstation. During the deposition process, the stirring rate was kept constant at 1000 rpm. After that, the catalyst was calcined under vacuum at 250 °C for 1 hour and then annealed in air at 350 °C for 4 hours. The loading mass is about 1.6 mg. The Co_1.5_Ga_1.5_O_4_, Co_2.5_Ga_0.5_O_4_, and Co_1.5_Al_1.5_O_4_ catalysts were synthesized following a process similar to that of the Co_3_O_4_ catalyst, with addition of Co(NO_3_)_2_·6H_2_O (0.05 M and 0.083 M), Ga(NO_3_)_3_·*x*H_2_O (0.05 M and 0.017 M), and Al(OH)(C_2_H_3_O_2_)_2_ (0.05 M) precursors. The geometric surface area of the samples was ∼0.25 cm^2^. In order to confirm the identity of these compounds, they were further characterized with XPS, XRD and SEM. SEM was performed using a Hitachi SU7000. XRD was performed using a Bruker D8; all materials were exposed to Cu-Kα radiation (*λ* = 0.15406 nm) and the data was collected with a point step of 0.02°. The XPS spectra in this study were analysed using the ThermoAvantage software. All XPS spectra were calibrated based on the position of the C1s peak in each spectrum and the Co2p peak convolution was performed using the same software as well. A Shirley background was used to determine the background of the XPS spectrum.

#### Electrochemical characterization

Electrochemical data were collected using a three-electrode system connected to an electrochemical workstation (Autolab PGSTAT302N) SCE and a carbon rod were used as reference and counter electrodes, respectively. 1 M KOH without saturated O_2_ was used as the electrolyte. Cyclic voltammetry (CV) measurements at 50 mV s^−1^ were performed for 3 cycles prior to recording linear scan voltammetry (LSV) at 5 mV s^−1^ for each sample.

#### Electrochemically active surface area (ECSA) determination

The ECSA of each catalyst was determined by measuring the electrochemical double-layer capacitance (*C*_dl_) of each catalyst from the scan-rate dependence of the CV plot. Four different CV measurements were performed at a potential window between 0.72 to 0.82 V (*vs.* the reversible hydrogen electrode) on each catalyst at scan rates of 20,30, 40 and 50 mV s^−1^ respectively. The *C*_dl_ was estimated at the average potential within this range by calculating the slope of the linear fit at that point. A specific capacitance (*C*_s_) of 40 μF cm^−2^ was used to calculate the ECSA of the catalyst using the following equation:
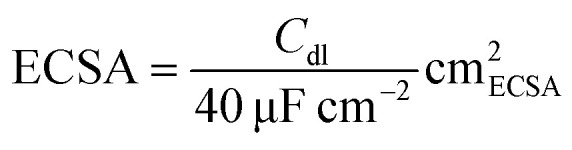


The LSV of each plot was subsequently normalized by dividing the current density in each plot with the calculated ECSA of each catalyst.

#### Potential calibration and *iR* correction

The potentials *versus* SCE (*E*_SCE_) were calibrated *versus* RHE (*E*_RHE_) using the following equation:
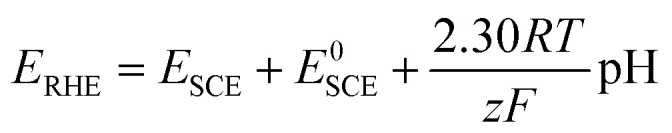
where *R* is the ideal gas constant (8.314 J mol^−1^ K^−1^), *T* is the temperature (298 K), *F* is the Faraday constant (96 485 C mol^−1^ electrons), *z* is the number of electrons (1 mol) transferred, *E*^0^_SCE_ is the standard potential of the SCE reference electrode that has been calibrated *versus* RHE. The pH is 14.

The potentials of the LSV were corrected by subtracting *iR*_S_, where *i* is the current measured at the corresponding potential and *R*_S_ is the bulk and solution resistances. The resistance *R*_S_ was calculated by fitting electrochemical impedance data in the 0.1–1 Hz range with the Randles circuit model. The *R*_S_ for each catalyst is given in Table S6 of the ESI.†

## Data availability

Data for this article that hasn't been included as part of the ESI† can be downloaded from: https://github.com/govlum/OER_HTS_Spinels.

## Author contributions

S. G. H. K., Z. Y. and A. A.-G. conceptualized this project. S. G. H. K. and Z. Y. planned the DFT high-throughput screens. S. G. H. K. performed the DFT calculations of both stages of the high-throughput screen with the guidance of Z. Y., S. G. H. K. and C. B.-G. implemented the random-forest and LASSO models to predict the binding energies of OER intermediates. N. W. and J. A. were involved with the synthesis and electrochemical characterization of the materials reported in this paper. C. B.-G. proposed experiments to structurally characterize the materials reported in this paper. S. G. H. K., C. B.-G. and C. H. S. were involved with interpreting the XRD and XPS results. S. G. H. K., C. B.-G. and Z. Y. were involved with writing the manuscript. S. G. H. K., C. B.-G., Z. Y., N. W., J. A. and A. A.-G. were involved with editing the manuscript.

## Conflicts of interest

There are no conflicts to declare.

## Supplementary Material

SC-015-D4SC00192C-s001
