# Supplementary material for: From computational screening to the synthesis of a promising OER catalyst
Source: Chem Sci. 2024 Jun 11;15(27):10556–70. doi: 10.1039/d4sc00192c (PMC11234821; doi:10.1039/d4sc00192c)
Supplement: SC-015-D4SC00192C-s001 [file SC-015-D4SC00192C-s001.pdf]

Electronic Supplementary Material (ESI) for Chemical Science.
This journal is © The Royal Society of Chemistry 2024

From computational screening to the synthesis of a promising OER catalyst – Supplementary Information

Sai Govind Hari Kumar, Carlota Bozal-Ginesta, Ning Wang, Jehad Abed, Jonathan Shan, Zhenpeng Yao, Alan Aspuru-Guzik*

Table S1: Performance of all four catalysts in this report

Compound	Overpotential at 10mA/cm^2 (mV)	Tafel Slope (mV.dec^{-1})
$GaCo_2O_4$	270	79
Co_3O_4	170	65
$Co_{2.5}Ga_{0.5}O_4$	220	56
$Co_{1.7}Al_{1.3}O_4$	193	77

Table S2: Compositional ratios of all elements of all materials explored in this report based on the ratios of the areas of peaks corresponding to $Co^{2+}$and Co^{3+}. Data obtained from XPS

Assumed Composition	Co^{2+} ratio	Co^{3+} ratio	True Composition
Co_3O_4	1	2	Co_3O_4
$Co_{2.5}Ga_{0.5}O_4$	1	1.5	$Co_{2.5}Ga_{0.5}O_4$
$Co_{1.5}Ga_{1.5}O_4$	1	1	$GaCo_2O_4$
$Co_{1.5}Al_{1.5}O_4$	0.7	1	$Co_{1.7}Al_{1.3}O_4$

Table S3: Double layer capacitance and ECSA of each catalyst studied in this report. The specific capacitance used to determine the ECSA is 0.04 mF

Catalyst	Capacitance (mF)	ECSA
Co_3O_4	282	7050
$Co_{2.5}Ga_{0.5}O_4$	163	4075
$Co_{1.5}Al_{1.5}O_4$	265	6625
$Co_{1.5}Ga_{1.5}O_4$	121	3025

Table S4: U values used for all DFT calculations on this report. All U values are taken from Ref. 13

Element	U-value
Sc	3.0
Ti	3.9
V	3.5
Cr	3.5
Mn^{2+}	5.25
Mn^{3+}	4.0
Fe^{2+}	4.0
Fe^{3+}	4.0
Co^{2+}	4.5
Co^{3+}	3.3
Ni^{2+}	6.0
Ni^{3+}	6.4
Cu	6.0
Zn	4.7

Catalyst (1M KOH)	Overpotential at 10mA/cm^2(mV)	Tafel Slope (mV.dec)	Reference
$Co_{2.5}Ga_{0.5}O_4$	220	56	This work
$GaCo_2O_4$	270	79	This work
Co_3O_4	170	65	This work
$Co_{1.7}Al_{1.3}O_4$	193	77	This work
Ni-Fe Boride	167	25	45
Amorphous NiFe	265	25	41
NiFe LDH/$NiFeS_x$	210	31	40
NiFe-OOH/Ni foam	250	36	52
Amorphous LaNiFe-OH	189	36	42
NiFe-LDH/NiCu	218	56.9	49
NiFe-LDH	195	40.3	50
Au/NiFe-LDH	237	36	54
Ni-Fe-O nanowires	244	39	48
NiFeSe/Ni foam	198	54	52
NiMoFe/Ni foam	257	53	52
NiFe-LDH/NiCoP	220	48.6	44
FeCoW/Ni foam	210	53	52
CoV-OOH/Ni foam	268	56	52
CoV-O/Ni foam	314	57	52
CoFeSe/Ni foam	195	63	52
CoPi	242	48	52
CoFePi	273	39.5	39
CoMnP	330	61	55
$Ni_xCo_{3-x}S_4$	160	95	47
$Co_3O_4/NiCo_2O_4$	320	84	59
RuO_2/CeO_2	350	74	46
RuO_2/NiO/Ni foam	250	50.5	51
NiFe-LDH(nanosheets)	300	68	53
RuO_2/Ni foam	283	53.4	51
IrO_2/Ni foam	333	55.6	51
Co_3O_4/RuO_2	250	55.4	38
Amorphous CoOx/RuO_2	270	69.6	43
Co_3O_4/Ppy/VC	330	105	57
Co_3O_4/Ppy/RGO	300	95	57
Co_3O_4 microspheres	400	100	56
Co_3O_4/CMC	290	71	58
Co_3O_4/Polyacrylamide	260	63	58
$NiFe_2O_4/Fe_3Ni$	262	39.5	9
$CoFe_2O_4$/C nanorods	240	45	10
$CuCo_2O_4$/C	288	64.2	11
$ZnFe_2O_4$/Ni foam	280	70	60
$NiCo_2O_4$	240	50	61

Table S5: Benchmarking analysis of catalysts explored in ths work with well-performing catalysts from other work

Table S6: Solution Resistances for each catalyst

Samples	Bulk/Solution Resistance (**Ω**)
Co_3O_4	2.86
$Co_{1.7}Al_{1.3}O_4$	2.93
$GaCo_2O_4$	3.02
$Co_{2.5}Ga_{0.5}O_4$	3.35

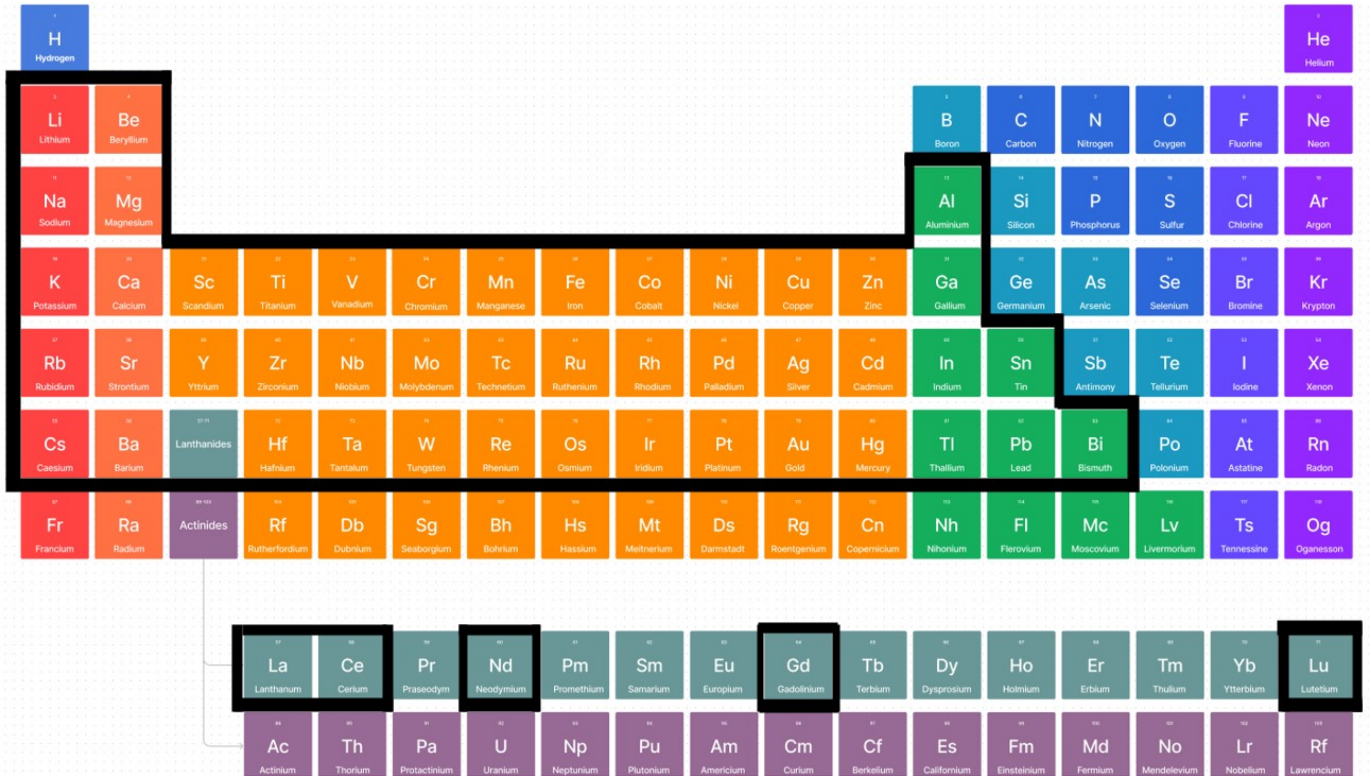

Figure S1: Periodic table of elements. The elements circled in black represent (apart from Tc) the elements examined this report. All the alkali, alkali earth, transition and post-transition metal elements were examined in this report. Some lanthanides (La,Ce,Nd, Gd and Lu) were also included as well, however, not all lanthanide elements could be included because the number of permutations of compounds would get unmanageably large.

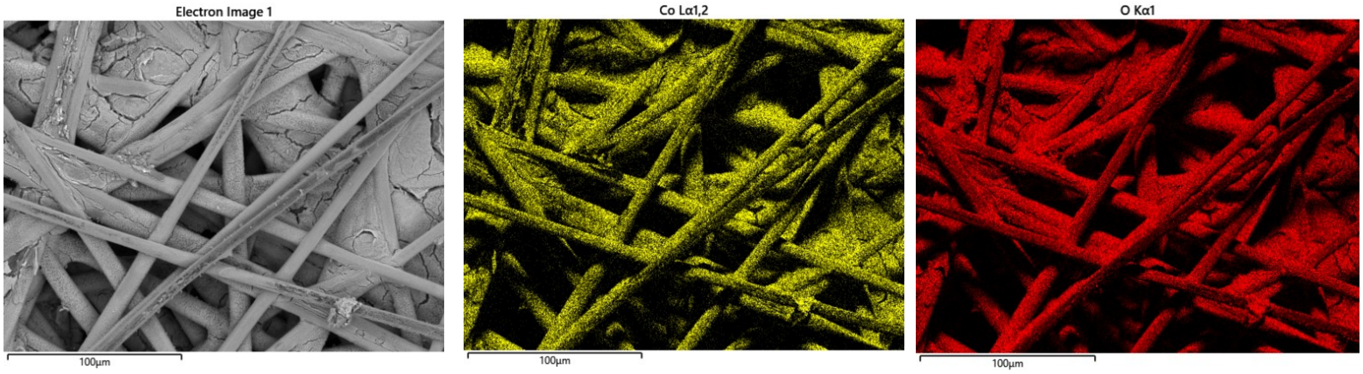

Figure S2: EDS Map of Co_3O_4

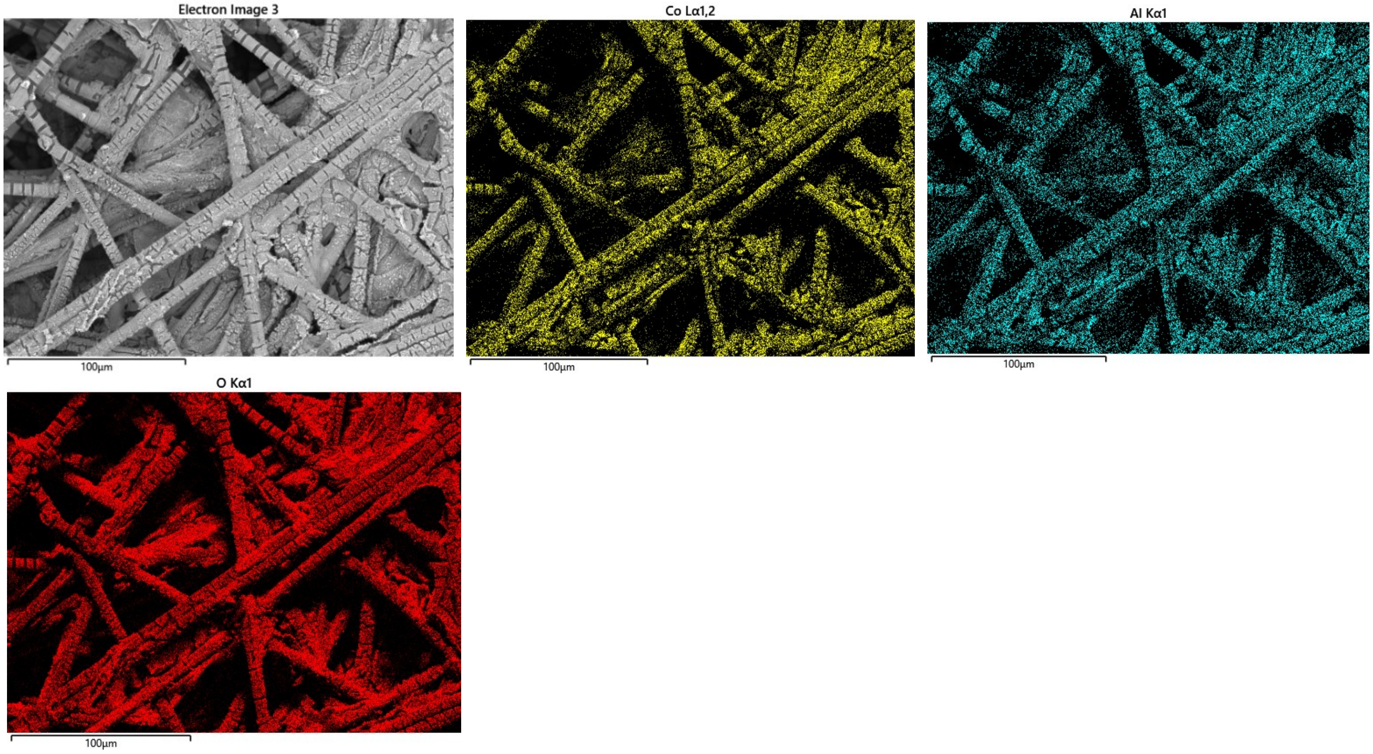

Figure S3: EDS Map of $Co_{1.5}Al_{1.5}O_4$

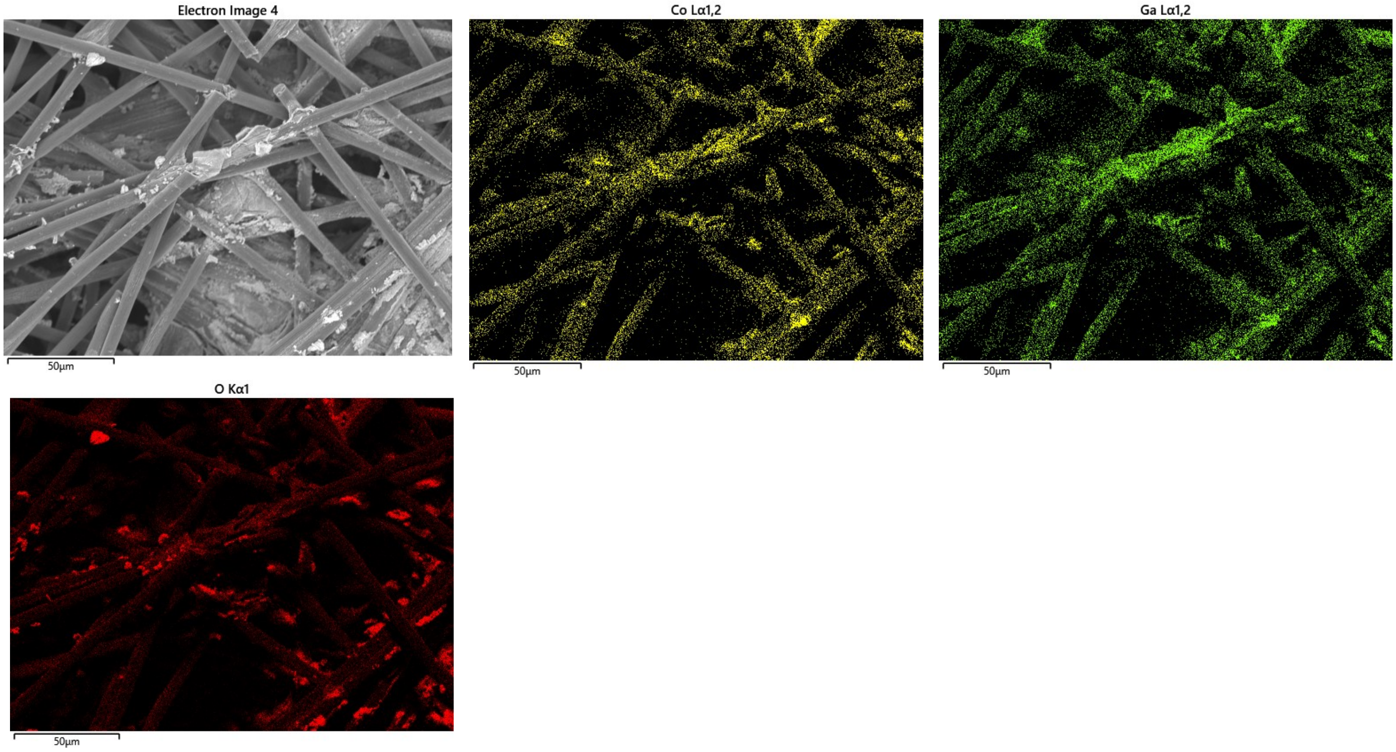

Figure S4: EDS Map of $Co_{2.5}Ga_{0.5}O_4$

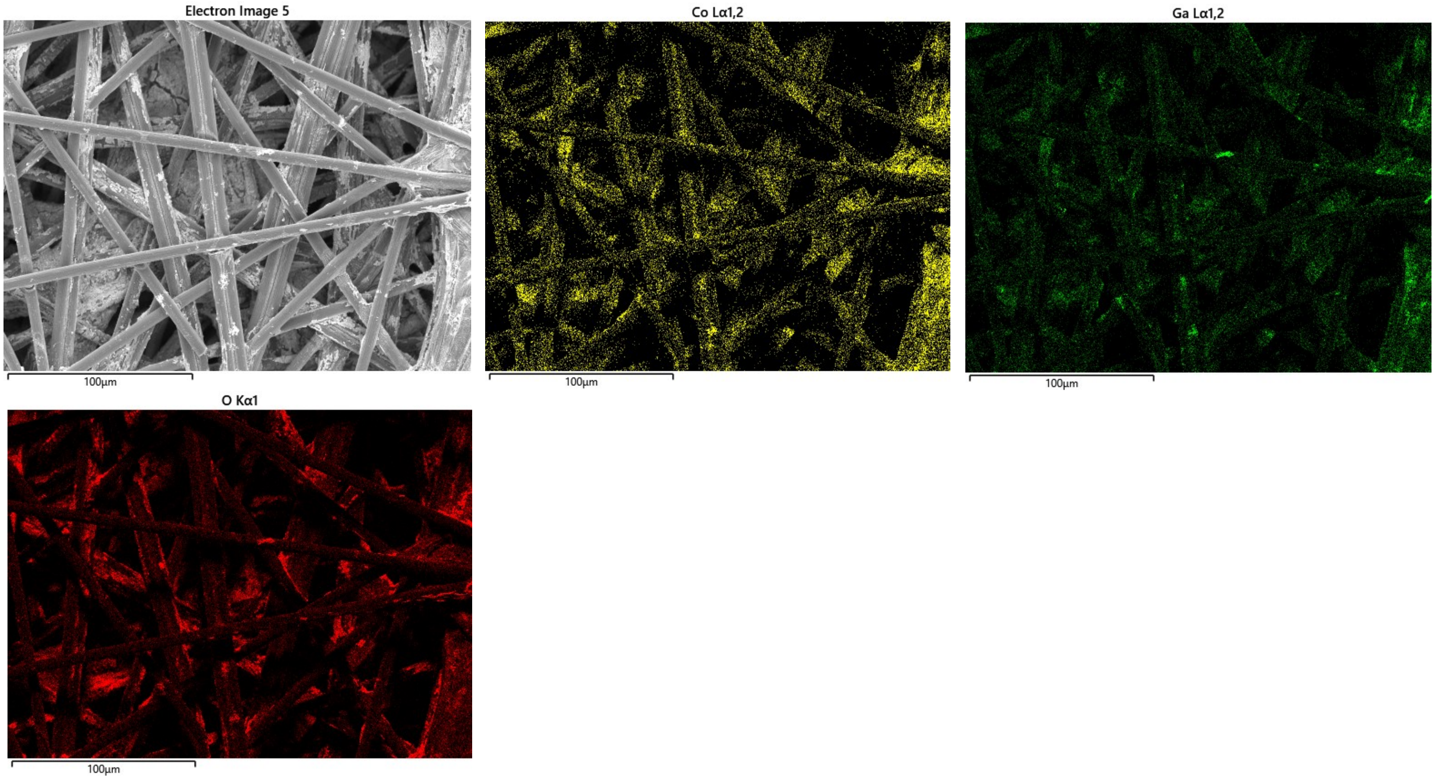

Figure S5: EDS Map of $Co_{1.5}Ga_{1.5}O_4$

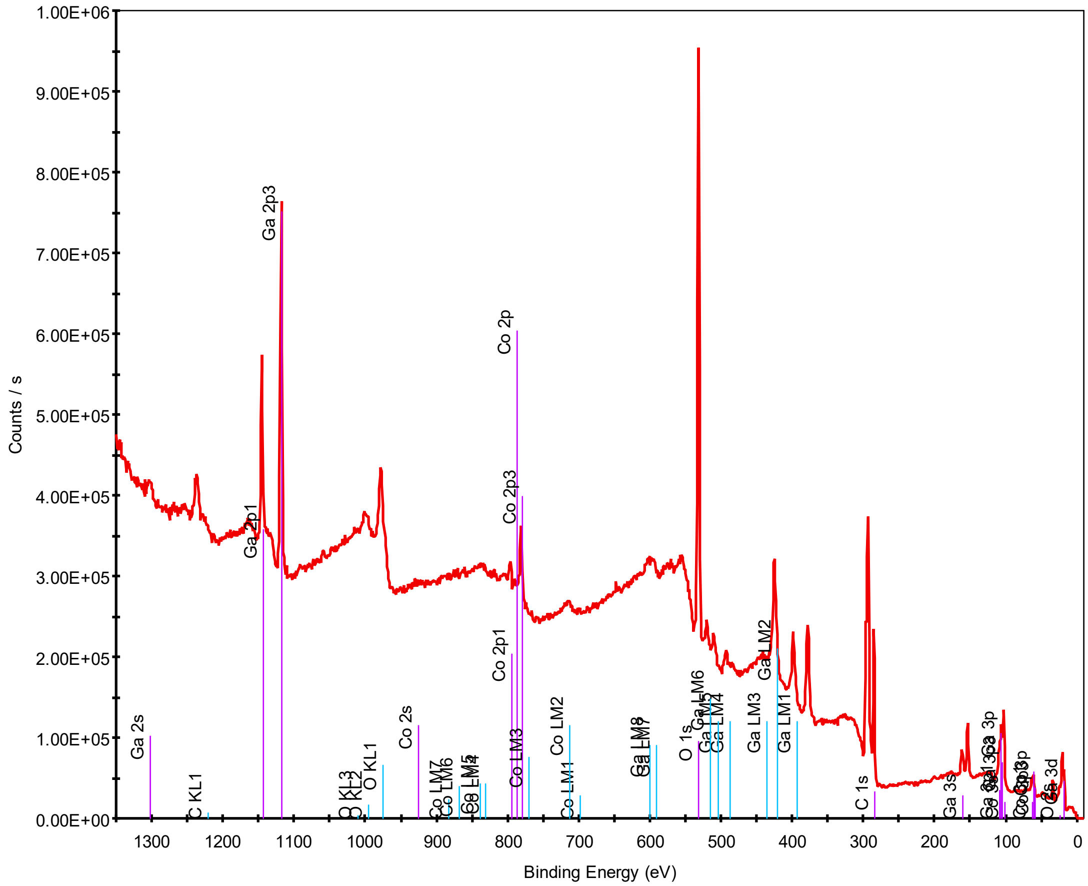

Figure S6: XPS Survey Spectrum of $GaCo_2O_4$

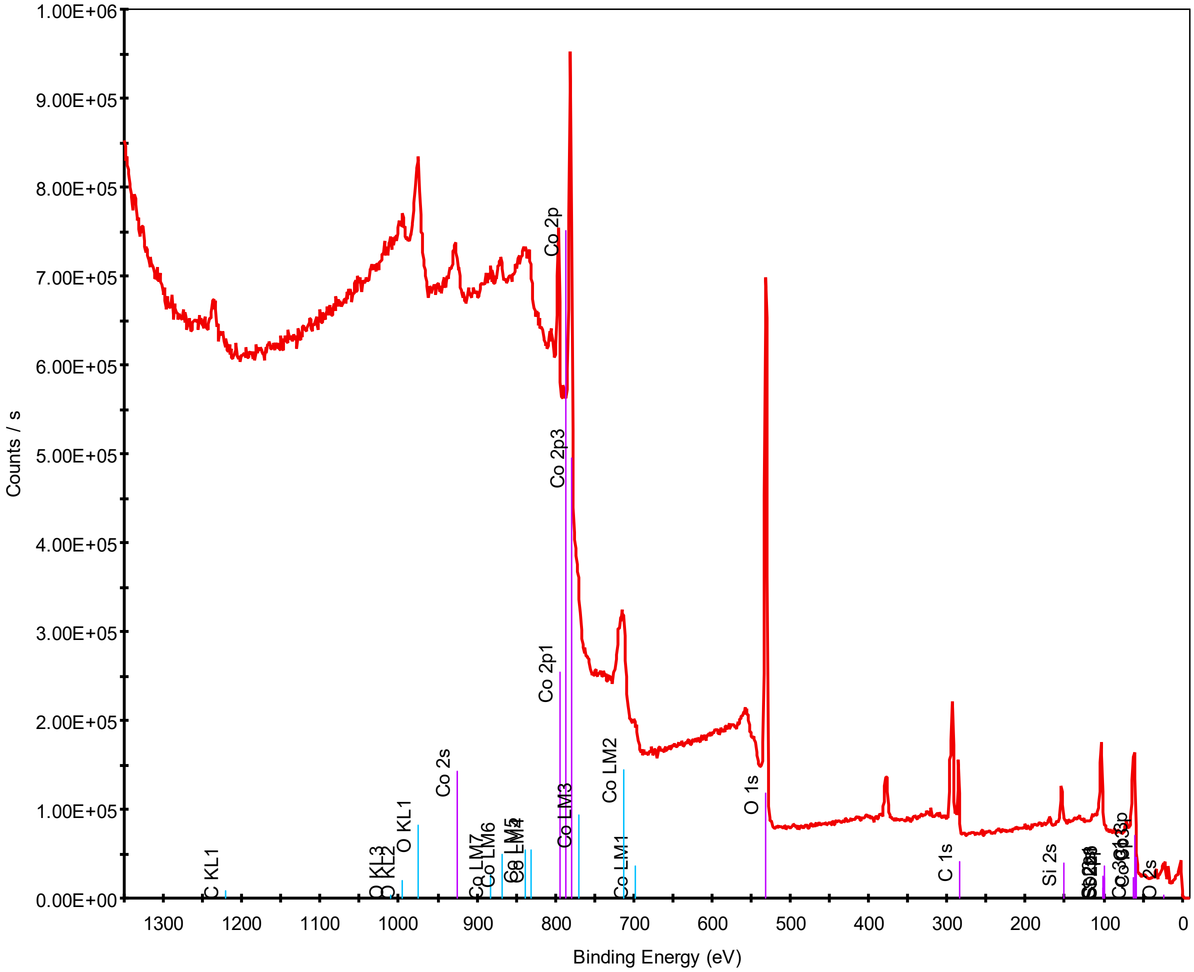

Figure S7: Survey Spectrum of Co_3O_4

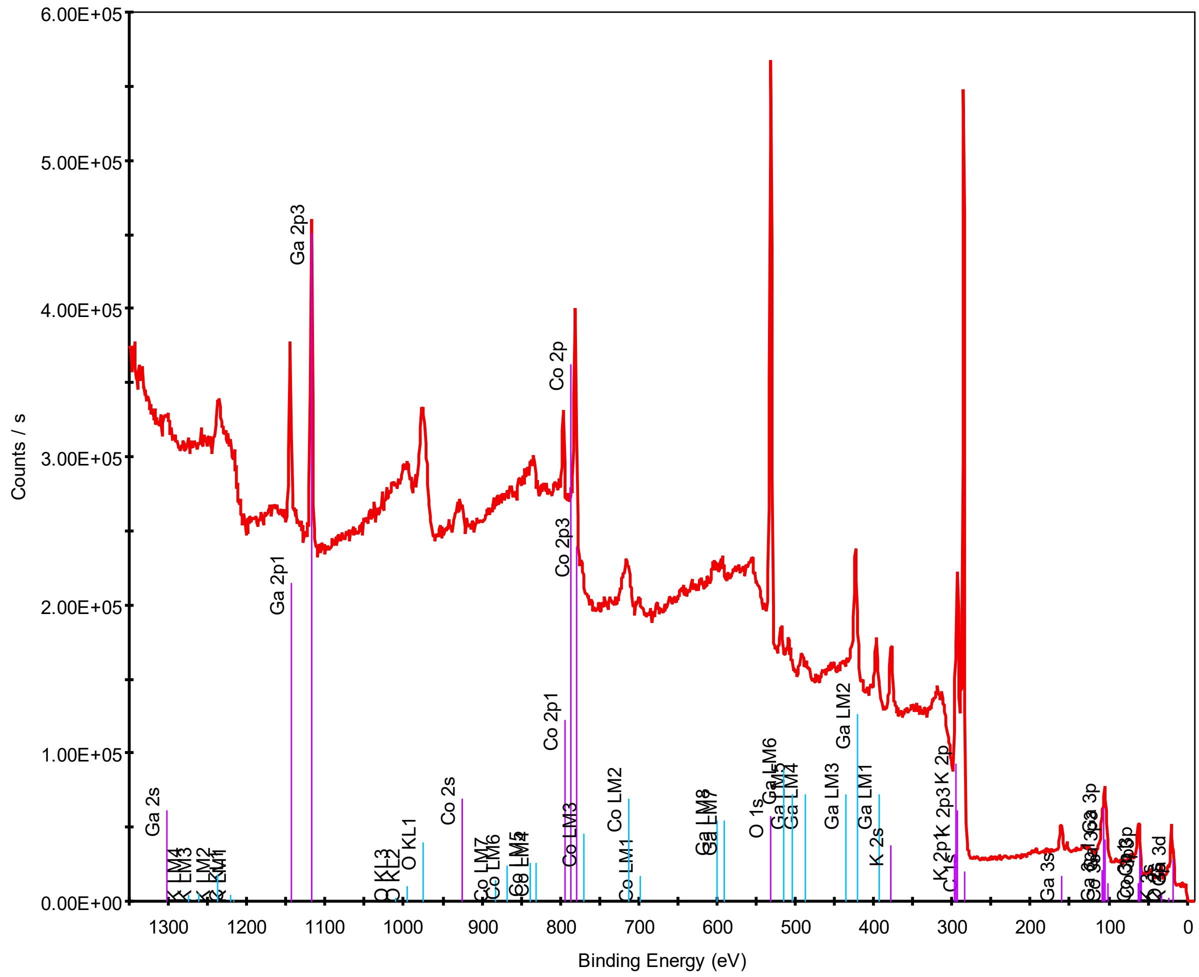

Figure S8: Survey Spectrum of $Co_{2.5}Ga_{0.5}O_4$

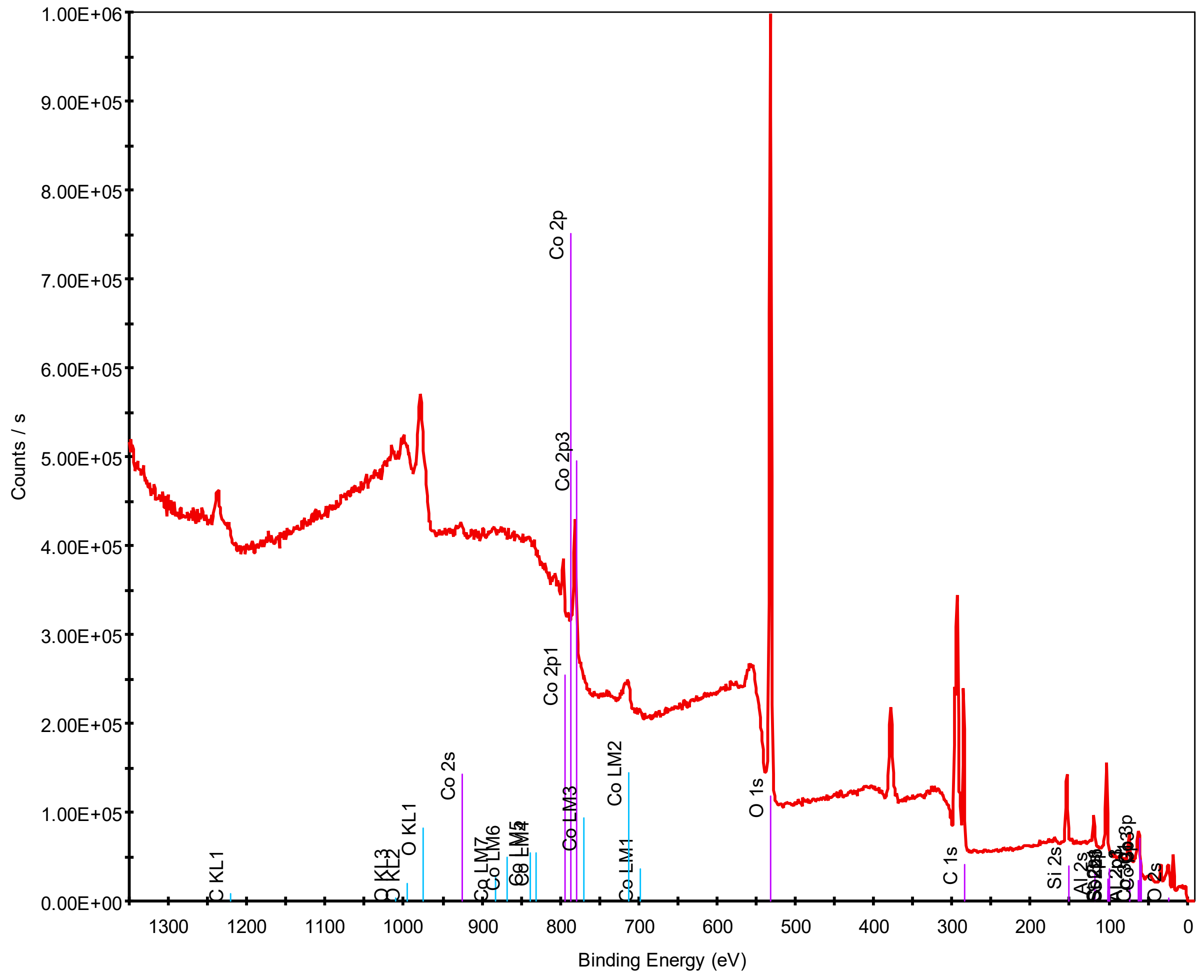

Figure S9: XPS Survey Spectrum of $Co_{1.7}Al_{1.3}O_4$

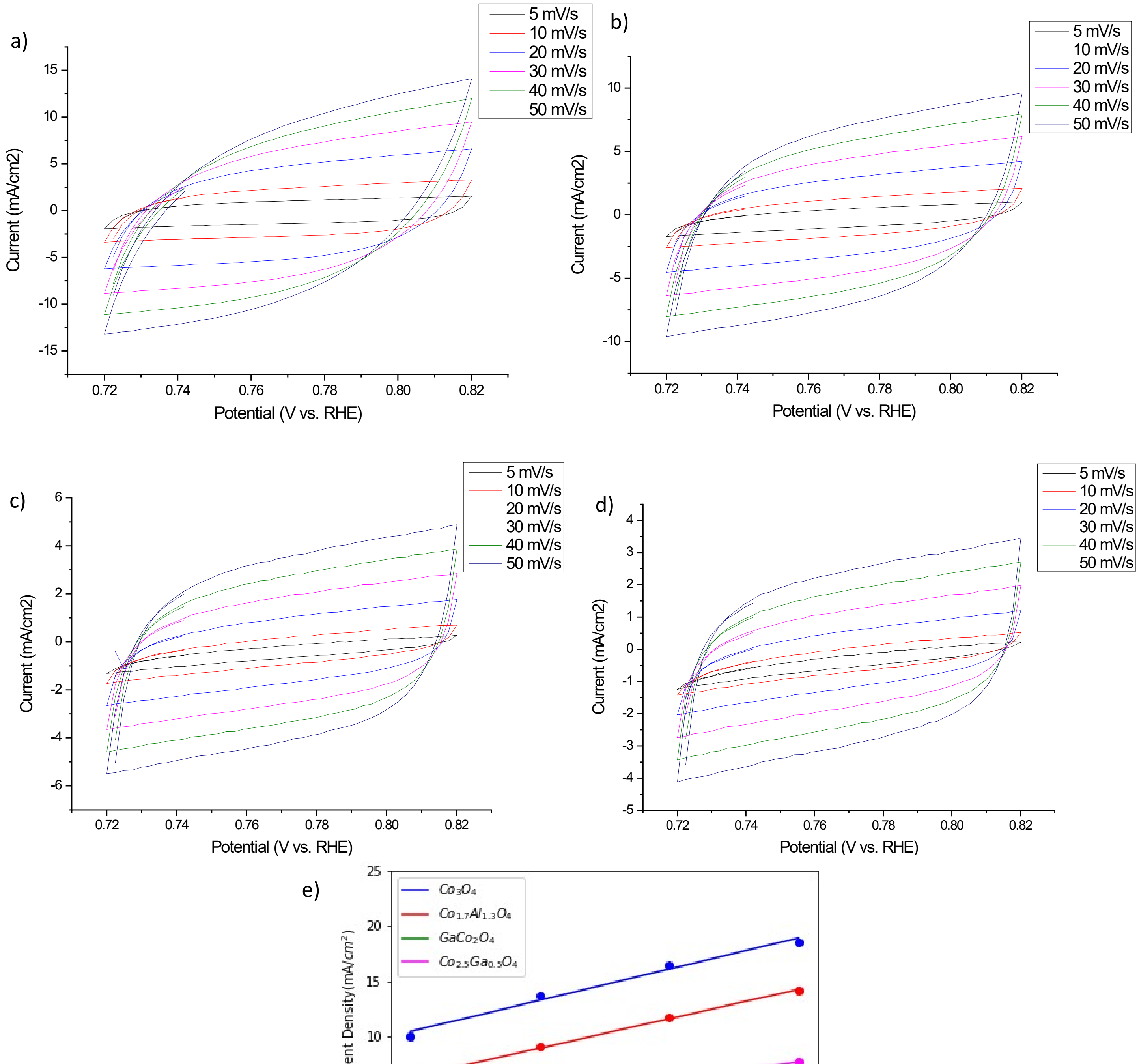

a)
5 mV/s
10 mV/s
20 mV/s
30 mV/s
40 mV/s
50 mV/s
Current (mA/cm2)
Potential (V vs. RHE)
b)
5 mV/s
10 mV/s
20 mV/s
30 mV/s
40 mV/s
50 mV/s
Current (mA/cm2)
Potential (V vs. RHE)
c)
5 mV/s
10 mV/s
20 mV/s
30 mV/s
40 mV/s
50 mV/s
Current (mA/cm2)
Potential (V vs. RHE)
d)
5 mV/s
10 mV/s
20 mV/s
30 mV/s
40 mV/s
50 mV/s
Current (mA/cm2)
Potential (V vs. RHE)
e)
Co_3O_4
$Co_{1.7}Al_{1.3}O_4$
$GaCo_2O_4$
$Co_{2.5}Ga_{0.5}O_4$
ent Density(mA/cm^2)

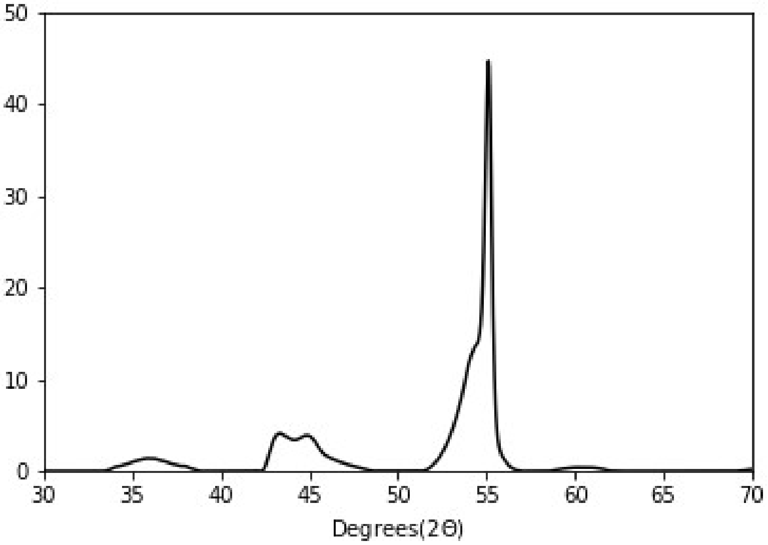

Figure S11: XRD spectrum of the carbon paper substrate

A)

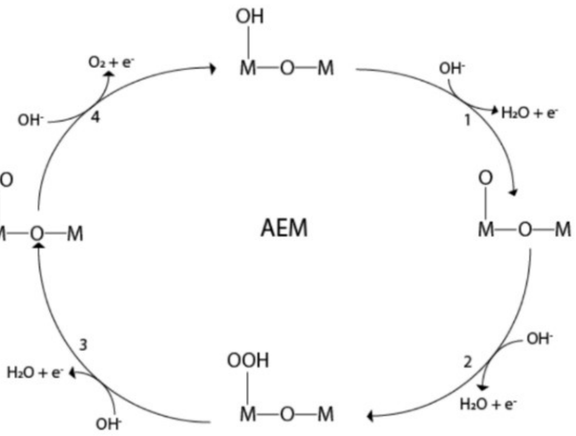

B)

$$\Delta G_1 = \Delta G_{O^*} - \Delta G_{OH^*} + k_B T ln a_{OH} - eU$$

$$\Delta G_2 = \Delta G_{OOH^*} - \Delta G_{O^*} + k_B T ln a_{OH} - eU$$

$$\Delta G_3 = \Delta G_{O_2} - \Delta G_{OOH^*} + k_B T ln a_{OH} - eU$$

$$\Delta G_4 = \Delta G_{OH^*} - \Delta G_{H_2O(l)} + k_B T ln a_{OH} - eU$$

Figure S12: a) The four-step AEM mechanism of water oxidation b) Free energy calculations of each step outlined in a) based on the computational hydrogen electrode model. ΔG_{O*}, ΔG_{OH*}, ΔG_{OOH*} and ΔG_{H2O} refer to the binding energies of O*, OH*, OOH* and H_2O^* on the surface of the material. k_B refers to the Boltzmann constant, T refers to the temperature, a_{OH} refers to the concentration of OH^- ions, e is the charge of an electron in eV and U refers to the applied potential. Calculations are performed with the assumption that a_{OH}= 1M and U = 0V.

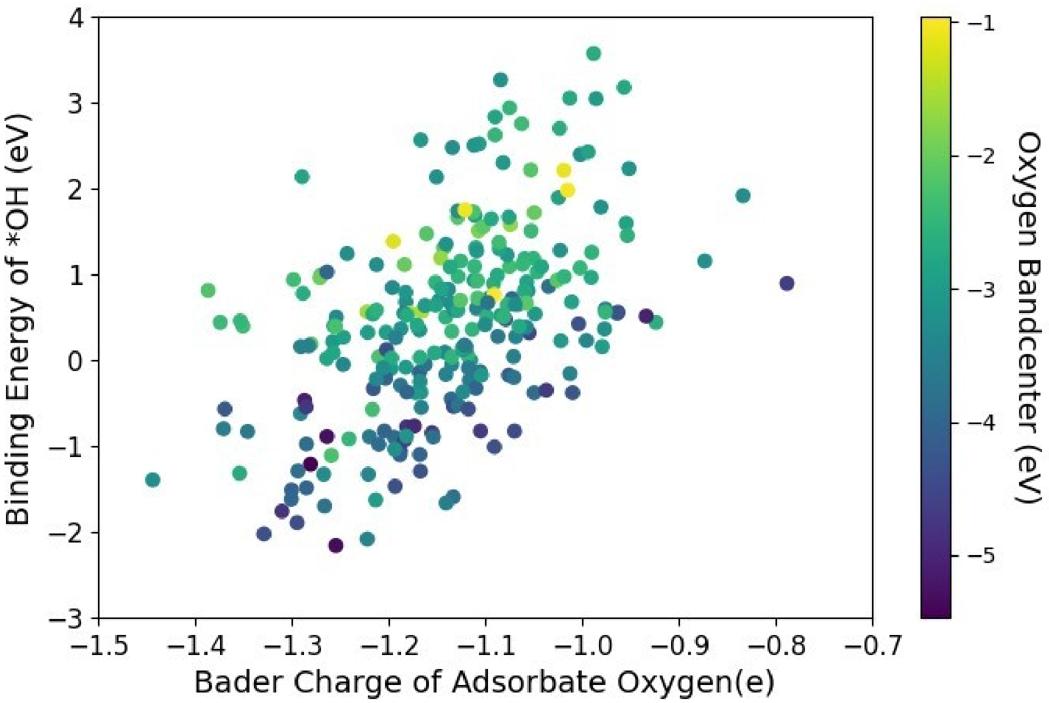

Figure S13: Bader charge of the adsorbate oxygen is plotted against the binding energy of the *OH intermediate. The colorbar represents the O2p bandcenter of adsorbate oxygen

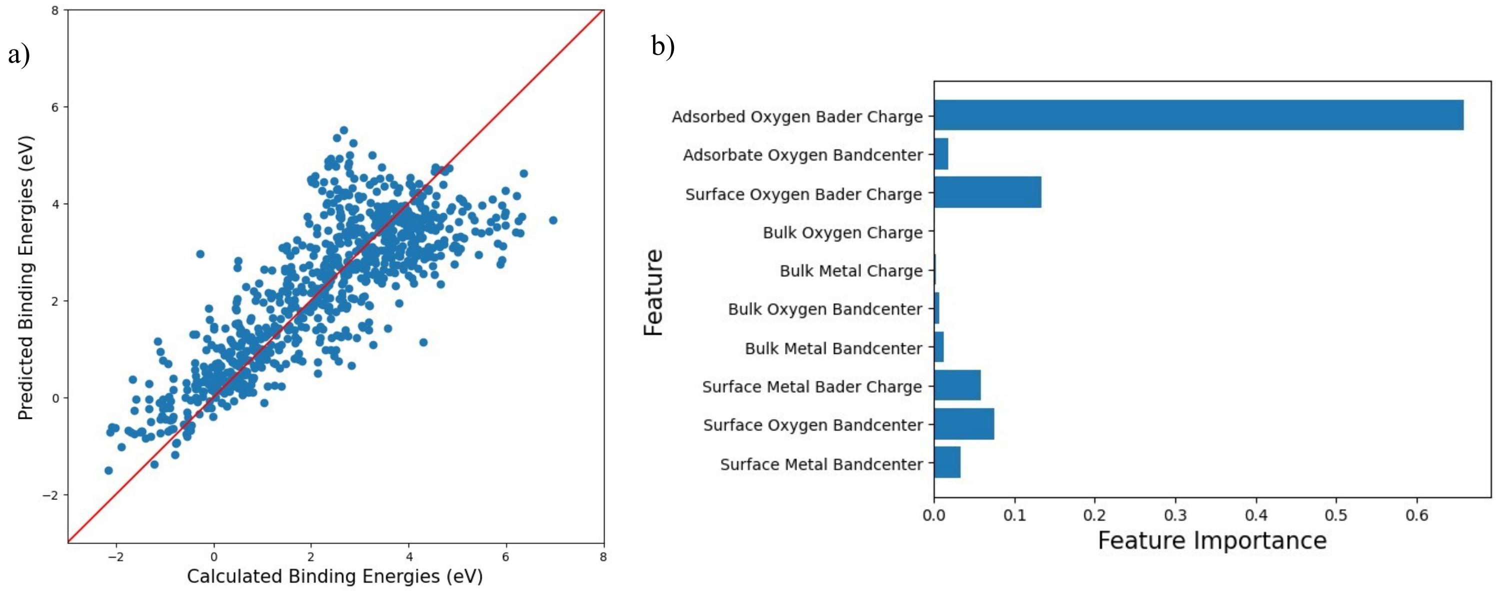

Figure S14: LASSO Regression of binding energies of *O, *OH and *OOH intermediates using the same eight calculated features. LASSO performs a lot more poorly than the random-forest model at predicting the energy of the *O, *OH and *OOH intermediates; with an R^2 value of 0.65 for the training set and an R^2 value of 0.72 for the test set. A) A graph to compare the predicted binding energies of the LASSO model with the calculated ones. The median absolute error is 0.56 eV for both the training and test sets. B) A bar graph that denotes the relative importance of the features that were inputs into the model. Just like in the random-forest model, the Bader charge of adsorbed oxygen was the single most important factor predicting the binding energies of the intermediates.
